# A forgotten collection: the Libyan ethnobotanical exhibits (1912-14) by A. Trotter at the Museum O. Comes at the University Federico II in Naples, Italy

**DOI:** 10.1186/1746-4269-8-4

**Published:** 2012-01-21

**Authors:** Antonino De Natale, Antonino Pollio

**Affiliations:** 1 Department of Soil, Plant, Environmental and Animal Production Sciences, University of Naples (Federico II), Via Università, 100, 80055 Portici (NA), Italy; 2 Department of Biological Sciences/Section of Plant Biology, University of Naples (Federico II), Via Foria, 223, 80139 Napoli, Italy

**Keywords:** Herbarium, North Africa, Libya, Traditional plant use, Medicinal plants

## Abstract

**Background:**

The Ethnobotanical Collection from the Libyan territories of the botanist Alessandro Trotter is included in the Oratio Comes Botanical Museum at the Faculty of Agraria at the University Federico II in Naples. Trotter explored different territories of Libya, mainly Tripolitania, between 1912-1924, collecting plant specimens and the drugs most frequently sold in the markets. The Libyan herbarium currently includes over 2300 sheets of mounted and accessioned plants. The drugs, mostly acquired by Trotter from Tripolitanian markets, were identified and packed in 87 paper sheets or boxes. Trotter added ethnobotanical information for each species when available.

**Methods:**

A database of the herbarium species and the drugs has been carried out, after a taxonomic update. Nomenclature has been revised according to the African flowering plants database and the World Checklist of selected plant families, and a comparison with currently available ethnopharmacological data from North African has been attempted.

**Results:**

In this study, ethnopharmacological data related to about 80 species of flowering plants and to 4 lichens are presented. The plants are mainly from Mediterranean or Sub-Saharan habitats and belong to 37 different families; Lamiaceae was the most cited family, with 10 accessions. Generally, the aerial parts of the plants are the most frequently used (28 species), followed by leaves (15 species), flowers and seeds (9 species), fruits (7 species) and hypogean organs (roots, rhizomes, tubers: 5 species). Plants were generally processed in very simple ways: infusion or decoction of the plants were prepared and orally administered or used for topical applications. A wide range of conditions was treated, ranging from mental disorders to skin affections. All the organs of human body are considered, but the pathologies of gastro-intestinal tract, respiratory system and those related to traumatic accidents were the most frequently mentioned. The comparison with the recent ethnopharmacological research in Maghreb and its neighboring countries reveals a high correspondence; almost all the plants cited by Trotter are still used in the folk medicine of at least one of the North African countries, and the therapeutic uses of each plant appear consistent over time.

**Conclusions:**

The information collected by Trotter is an important contribution to tracing plant utilization in Libyan folk medicine over the last century.

## Background

**Figure 1:**
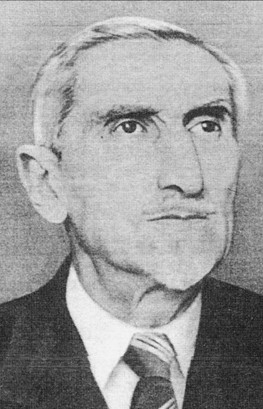
**A portrait of Alessandro Trotter**.

**Figure 2:**
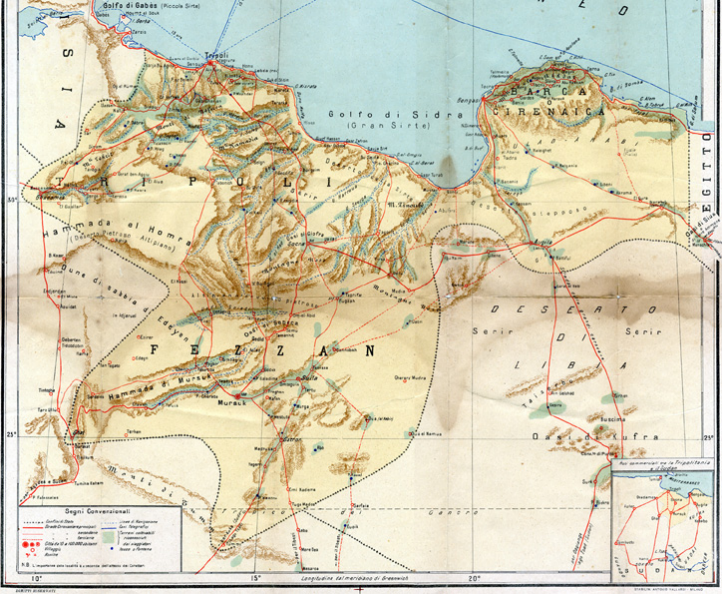
**Geographical map of Libya in 1912 **[4].

The Orazio Comes Botanical Museum at the Facoltà di Agraria dell'Università Federico II di Napoli encompasses the Historical Library, the Herbarium Porticense (PORUN), the Mycological Herbarium (POR) and wood collections (Xilotomoteque and Xiloteque). These wide-ranging collections were created in the course of the 19th and 20th Centuries and were kept in different sections of the Faculty of Agraria. Only recently was the Polo Museale delle Scienze Agrarie instituted, with the aim of unifying all the collections under a common structure. While the definitive building is under re-construction, the collections of the O. Comes Museum have been temporarily transferred to a provisional site. This on-going reorganization provided an opportunity for a careful examination of the items from each collection, including the archives, which document the work of various botanists from the early 1600s to 1940 [1-3]. One of the most interesting results of this work was the rediscovering of the Botanical Collection from the Libyan territories of the botanist Alessandro Trotter (Figure 1). A. Trotter was born on 1874 in Udine, Italy. In 1899, he graduated with a degree in Botany from the University of Padua, under Professor A. Saccardo, who accepted him as his assistant immediately after his graduation. A. Trotter remained at the University of Padua until 1902, when he was entrusted to teach Plant Pathology at the School of Enology of Avellino, and, subsequently, at the Agronomy Faculty of Portici, Naples, where he worked until 1949 as full professor of Botany. Trotter's scientific career reflected his broad interest in Botany. Since his beginning at the University of Padua, he demonstrated a strong attitude towards the floristic, with a specific interest in mycology, but he was also committed to the study of horticultural species, particularly those from South Italia. On behalf of the Italian Government, from 1912 to 1924, Trotter participated in different expeditions in Libya [4] (Figure 2), mainly in Tripolitania [5-7]. The results of Trotter's work in this region and, to a lesser extent, in other Libyan territories was a collection of plant specimens and a *repertorium *of the drugs he bought in the markets of Tripolitania, together with an archive of information, field notes, and photographs. Some of his findings were published in two reports [8,9], which are unavailable to the current scientific community. Over time, Trotter's materials were scattered throughout different rooms of the Botany Department and merged with other accessions belonging to different collections. The organization of the new museum offered the possibility of grouping Trotter's exhibits in one collection, which represents a unique source of ethnobotanical information, dating back to a century ago, on a scarcely known geographical area of North Africa. It has been stressed that there are only a few places in the world for which diachronic data for ethnopharmacological purposes are available [10]. Recent efforts on this subject have been recently presented for Estonia [11] and Poland [12,13], but old ethnobotanical data are very scarce for the African continent [14,15].

The aim of this paper is to report the data on plants used in folk medicine of Tripolitania and neighboring regions collected by Trotter during his expeditions in Libya and to present a preliminary description of the exhibits now available in the Libyan ethnobotanical collection of the Orazio Comes Museum.

## Methods

### Geographical description of the study area

North Tripolitania is dominated by the Jaffar Plain, which slopes downward to the west and upward to the highland territories (Jabel Nafusa), which in turn continue eastward with a belt of hills around Tarhuna and Homa [16]. The vegetation is dominated by steppes, which cover a zone extending from the coast west of Zamiya to a large part of Jaffara Plain, the Jabal Nafusa, and eastward to the Misurata region [17]. The soils around Tripoli, extending to central Jaffara, are rich in organic compounds, but the soils are poor and sandy in the rest of North Tripolitania and rocky in Jabal Nafusa. In coastal Tripolitania and in the Jabal Nafusa highlands, the climate is Mediterranean, with long, dry and hot summers, whereas in central and eastern Jaffara and in inland Jabal, the climate is semi arid, and drought occurs frequently [18]. At the beginning of 20th century, agriculture was concentrated in the oasis of the coastal territories and in part of the Jabal Nafusa plain. In the rest of the region, pastoralism (sheep, goats and camels) was the most common land use, together with transhumant cereal cultivation. The areas of mixed cereal cultivation and pastoralism were held by seminomadic groups through a combination of private and collective tenure [16].

### History of the Trotter Libyan Collections

After his first expedition to Libya (1912), Trotter assembled a collection of plants primarily from North Tripolitania. In the subsequent trips, he gathered specimens from other regions of Libya as well [5-7].

The Trotter Collection, now located in the Orazio Comes Museum, features herbarium samples, drugs, historical photographs, and manuscripts.

**Figure 3:**
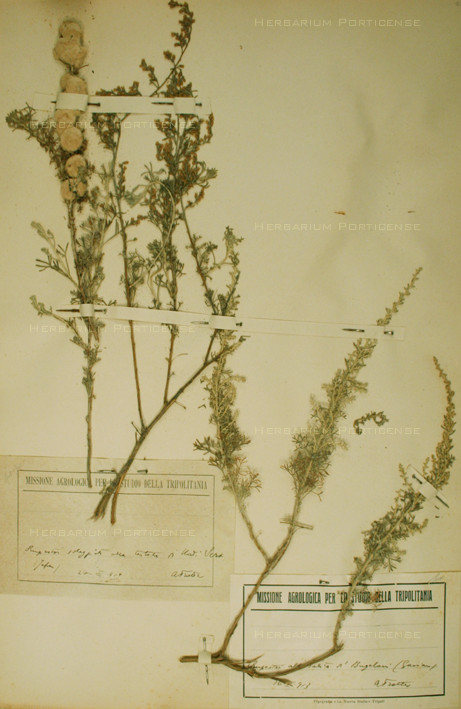
**Herbarium specimen from Trotter collection, *Artemisia herba-alba *Asso**.

The Libyan Fanerogamic collection currently includes over 2300 sheets of mounted and accessioned plants. The herbarium is organized alphabetically by family, and each herbarium sheet measures 43 × 28 centimeters (Figure 3). Paper specimen labels are attached to the right corner of the herbarium sheet. Loose parts or fragments of plant organs are included in envelopes attached to the herbarium sheet. Each voucher specimen was annotated by Trotter with the current taxonomic name at the time, the Libyan location from which the specimen was acquired, and the collection date. Additionally, Trotter added ethnobotanical information for about 80 species, which have now been grouped in a specifically designed archive.

**Figure 4:**
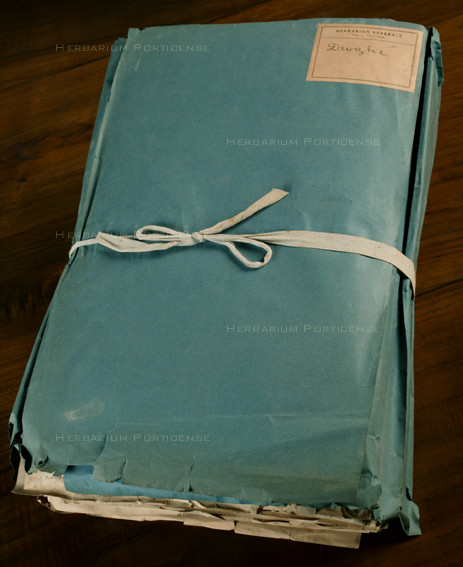
**A drug package**.

**Figure 5:**
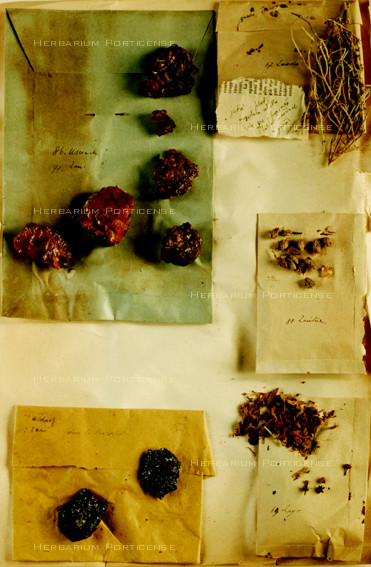
**Paper sheets and boxes containing the drugs**.

**Figure 6:**
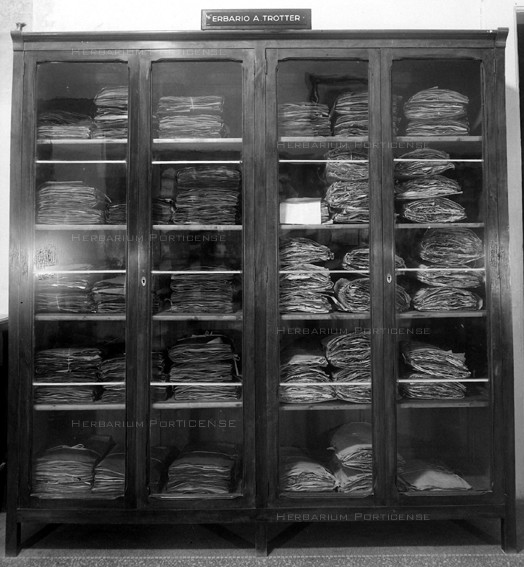
**The original wooden cabinet of Trotter Herbarium**.

The drugs (Figure 4), prevalently acquired by Trotter from Tripolitania markets, were identified and packed by Trotter in 83 paper sheets or boxes (Figure 5), which were stored in a wooden cupboard (Figure 6). Included in the collection were some unidentified drugs, corresponding to n. 74 and 83.

Recently, a database of the herbarium species and drugs has been carried out, and, subsequently, a taxonomic update has been attempted. The nomenclature has been revised according to the African flowering plants database [19] and the World Checklist of selected plant families [20].

## Results

Italian colonization of Tripolitania began in 1911 without a preliminary assessment of the land: for this reason, geographical expeditions in Libyan territories were undertaken between 1912 and 1916, with the participation of various specialists. The first expedition [5] had the task of carrying out mineralogical and agronomic studies on Tripolitania, and Trotter was the agronomist of the expedition. During his residence in Libya, he studied the flora (algae, fungi, and higher plants, including cultivated species) of Tripolitania. He was also greatly involved in the collection of information on plant uses from the local people. This interest was driven by practical needs, as plants used by Libyans could have potential economic benefits to emigrant settlers. In this paper, we have concentrated our attention on the medicinal uses of Libyan plants collected by Trotter. In Table 1[21-45], these ethnopharmacological data are compared with the recent available information from Libya and other neighboring countries.

**Table 1:** Plants used in Libyan folk therapy at the beginning of XXth Century according to Trotter data.

Botanical name	Local names	Part(s) used	Description of uses	Present uses in Libya and neighboring Countries
*Aaronsohnia pubescens *(Desf.) K. Bremer et Humphries (PORUN - TTF2300) ASTERACEAE	*uàs-uàsa *(arab); *uscescuane *(tuaregh)	Leaves	Grounded and eaten against gastro-intestinal aches	The whole plant in infusion is used for gastro-intestinal troubles and kidney stones. It is collected and sold in the markets of South Algeria [21]
*Achillea maritima *(L.) Ehrend. et Y.P. Guo (PORUN - TTF2318) ASTERACEAE	*agbita, sciba *(Algeria)	Aerial part	Sold as a febrifuge and emmenagogues	In Marmarica, it is known as medicinal plant [22]. In North African countries, the flowering branches are considered a febrifuge, emmenagogue, tonic, and taenifuge [23]
*Ajuga iva *(L.) Schreb. (PORUN - TTD52; TTF1411) LAMIACEAE Additional file 1	*sciandagúra *(arab); *assaron*? (Cirenaica); *tletúl teelscín *(berber)	Aerial part	A cold infusion is anthelminthic; mixed with other components, against pulmonary affections	In Marmarica, the plant is considered medicinal [22]. A decoction of the aerial parts is administered against rheumatic pains, and as a carminative and stomachic. Aerial parts are also used as incense in ritual practices [24]. In Morocco, the hot infusion is considered antidiabetic [23]; also used as an anthelmintic and for intestinal disorders [25]. Crushed leaves and seeds are smoked for their narcotic effects [26]
*Aloë vera *L. (PORUN - TTF2331) ASPHODELACEAE	*sabbàr*	Leaf juice	Medicinal properties (not described)	The transparent gel from leaf pulp is used as a vulnerary and laxative in different African countries [27]
*Alpinia officinarum *Hance (PORUN - TTD17) ZINGIBERACEAE Additional file 2	*cúlgan*, *cúlgian*	Rhizome	Tonic	Drug imported from East Asia and used in Egypt and Morocco, used as an antitussive and stimulant [22,25]. A preparation from the root is used for rheumatism and sexual impotence [24]
*Arbutus unedo *L. (PORUN - TTF895) ERICACEAE	*sc'meri *(arab); *linz *(Algeria); *isisnu*, *sciscnu *(berber)	Bark of roots and leaves	Astringent	The decoction of leaves or raw fruits to treat kidney diseases [26]
*Artemisia arborescens *L. (PORUN - TTD51) ASTERACEAE Additional file 3	*sézeret Marian *(arab); *scih*	Young shoots, flowers, leaves	A decoction against intestinal affections	In Morocco, the plant is considered anthelmintic, aperitive, diuretic, emmenagogue and abortive [25]. A leaf infusion to treat common colds, vertigo, and helminthiasis. Powdered leaves are externally applied for skin infections and wrinkles [24]
*Artemisia campestris *L. subsp. *variabilis *(Ten.) Greuter (PORUN - TTF484) ASTERACEAE	*sc'ahâl *(arab); *togoft*, *tegoft*, *taghert*, *tâghiat *(berber); *teghoch *(tuaregh)	Flowers, leaves	Anthelminthic, known as dua lehnâsc	In Algeria, it is used as a substitute for *A. absinthium *[23]. In Tassilli N'ajjer (South Algeria), aerial parts and flowers are used for post-partum care, emmenagogue, analeptic, and antispasmodic. Also used as an anthelminthic, for stomach and liver affections. Vulnerary [28]
*Artemisia herba-alba *Asso (PORUN -TTF482) ASTERACEAE Figure 3	*scih *(arab); *aghares *(berber); *azezzeré*, *zezzeri *(temahac)	The whole plant and the inflorescence	In the Fezzan, the dried plant is ground and used as a stomachic	In Marmarica region, it is known as fodder and as a medicinal plant [22]. A potion of the plant is drunk in North Sahara against digestive troubles, as an anthelmintic, and to treat eye affections and obesity [29]. A branch decoction is used for rheumatic pains and helminthiasis [24]. In Morocco, the plant is administered against gastrointestinal affection, as an antiseptic, anthelmintic, antidote to poisons, hypoglycemiant and emmenagogue [25]
*Asphodelus refractus *Boiss. (PORUN - TTF2304) ASPHODELACEAE	not reported	Leaves	Diuretic	Roots, shoots and leaves are used from Egypt to Morocco as a tonic and stomachic, against headache, liver affections rheumatisms, and for treatment of syphilis [23]
*Balanites aegyptiaca *(L.) Del. s.l. (PORUN - TTF2302) BALANITACEAE	*hagilidi*, *heglig *(arab); *tebôrac *(temahac); *tsciaisciot *(Tuat); *addua *(Haussa)	Young leaves, pulp of fruits	Leaves to clean sores; pulp fruit used in spleen diseases and to kill Guinea worms (*Vena medensis*)	The leaf decoction is used in the central Sahara (Algeria) and in other North African countries, as an anthelminthic, against skin affections, and as a vulnerary. Also administered to cure stomach, liver, pulmonary and spleen affections [23,28]. In Sudan, the fruit is administered as a purgative, a bark decoction against jaundice, and branch fumigation against rheumatisms [30]. Also used as purgative and for bilharzias [31]
*Boswellia sacra *Flüeckiger (PORUN - TTD39; TTF2305) BURSERACEAE Additional file 4	*lúban*	Resin of stem	The yellowish granules of the resin are burnt and chewed for their aphrodisiac properties	The species grows in South Arabia and is known by the Arabians as "maghrayt d'sheehaz". The resin is traditionally sold in African markets as a disinfectant and also used in the preparation of cosmetics [32]
*Brassica napus *L. s.l. (PORUN - TTF2308) BRASSICACEAE	lé*f*t (arab); *afrân *(temahac)	Seeds	Medicinal properties (not described)	Included among Libyan medicinal plants as an emollient (roots) [33]
*Capparis orientalis *Veillard in Duh. (PORUN - TTD7; TTF101) CAPPARACEAE Additional file 5	*cábbar *(arab); *tilult *(berber)	Leaves	Near Tripoli, a decoction of the plant is mixed with other herbs as a stomachic	In North Sahara, it is administered as a pain-killer, mainly to treat toothaches, and against rheumatisms [29]. In central Sahara (Tassili N'ajjer, Algeria), a poultice of fresh leaves is topically applied against rheumatism pains and headache [28]. Flowers and fruits are macerated to treat rheumatism [26]. An infusion of root bark is used as a cholagogue [24]
*Citrullus colocynthis *(L.) Schrad. (PORUN - TTF2316) CUCURBITACEAE	*handel*, *handla *(arab); *taghillilut *(berber); *tassellet *(mezabita); *alched*, *hagi *(tuaregh)	Seeds	Seed infusion against the viper bites	Roots are used as an abortive. In Morocco, it is considered as a antiepileptic, anthelmintic, aphrodisiac, and hypoglycemiant. Also administered against gonorrhea and tinea [25]. Moreover, the fruit, broken into small pieces, is used to protect woolen clothing from moths [21]
*Coriandrum sativum *L. (PORUN - TTF2313) APIACEAE	*cússbur*, *cóssbor *(arab)	Fruits	A syrup made from the fruit used against pulmonary affections	Aphrodisiac, anti-inflammatory, tonic. Seed and leaf decoction used against kidney stones, intestinal pains, insomnia, and scurvy [25,26]. Fruits are placed onto fire to remove bad spirits [24]
*Cuminum cyminum *L. (PORUN - TTD9) APIACEAE Additional file 6	*chemún ahdar *(arab); *azcar *(tuaregh)	Fruits	Chewed against stomach disorders	A milk infusion of fruit is administered against gastric pains and as an intestinal antiseptic, carminative and sudorific [24,25]. Also used in veterinary medicine [23]
*Cupressus sempervirens *L. (PORUN - TTD60; TTF854) CUPRESSACEAE Additional file 7	*arz*, *sirùa *(arab)	Seeds	An infusion against cough and heart diseases	In Libya, Morocco and Tunisia, the cones are considered antidiarrheal, antihemorrhagic, astringent, diuretic, expectorant, and sudorific [23]. In the El-Jabal El-Akhdar region (Libya), leaves and cones are administered in different ways against asthma, piles, and vaginal discharge [34]
*Curcuma longa *L. (PORUN - TTF2320) ZINGIBERACEAE	*córcob*	Roots	Medicinal properties (not described)	In Morocco, it is used as a digestive, stimulant, for blood diseases, and against amnesia [25]. The powdered rhizome is taken orally as a condiment, tonic, calefacient and digestive [24]
*Cymbopogon schoenanthus *(L.) Spreng. s.l. (PORUN - TTD79) POACEAE Additional file 8	*led'her *(Mizda, arab); *bu'rucûba*, *semmad *(Algeria); *lemmed*, *tiberrimt*, *leberint *(Algeria)	Basal part of the plant	Fumigation against a kind of influence called ahón	The plant is used in different countries of North Africa as an antihirheumatic, diuretic, emmenagogue, and febrifuge [23]
*Cynodon dactylon *(L.) Pers. (PORUN - TTF1127) POACEAE	*négem *(arab); *tobbalt *(berber); *asezmir *(mezabita); *oscerar *(temahac)	Whole plant	The decoction is a diuretic	Diuretic [25]. Root decoction against stomach disorders [26]. A decoction of the rhizome or aerial part against rheumatisms, kidney stones, uterine and menstrual pains, and abortion [24]
*Cyperus rotundus *L. s.l. (PORUN - TTF2307) CYPERACEAE	*giaâd *(arab)	Tubers	A water potion against heart diseases	North African Countries. The tubercles are used to cure a wide range of affections [23] In Sudan, a tuber decoction is used to treat stomach troubles and as an anthelmintic [30]. Stem galls mixed with leaves of *Lawsonia inermis *are powdered, kneaded with water and applied as a hair tonic [24]. The tubers are used to increase body weight [21] In Morocco, the plant is considered to be a cosmetic and aromatic and is used in hair care [25]
*Elettaria cardamomum *(L.) Maton (PORUN - TTD8) ZINGIBERACEAE Additional file 9	*cacúla*	Fruits	Fruit is grounded and mixed with honey into a tonic	Powdered seeds are used in a preparation administered as an aphrodisiac, digestive, and stimulant. Seeds are also used as a condiment [24,25]
*Ephedra alata *Decne. s.l. (PORUN - TTF2310) EPHEDRACEAE	*alenda *(arab); *timatrat *(temahac)	Seeds and young shoots	Medicinal properties (not described)	Used in Algeria, Libya, and Morocco as an anthiasthmatic, anti-hypertensive, astringent, and depurative as well as used against headaches and for treatment of pulmonary affections [23]
*Euphorbia guyoniana *Boiss. et Reut. (PORUN - TTF918) *Euphorbia paralias *L. (PORUN - TTF922) EUPHORBIACEAE	*lebbîn *(arab)	Latex	Topical application against viper bites	Different *Euphorbia *species are used in Morocco to treat skin diseases [26]
*Ferula marmarica *Asch. et Schweinf. (PORUN - TTD22) APIACEAE Additional file 10	*fassuch*	Gum resin	Medicinal properties (not described)	Source of gum ammoniac [35]
*Globularia alypum *L. s.l. (PORUN - TTF2077) GLOBULARIACEAE	*zréga*, *zrga *(arab); *taselrha*? (berber); *tidi*-*n*-*tenet *(tuaregh)	Leaves and branches Aerial part	The decoction is used as a laxative, against intermittent fevers and topically in the cure of furuncles	In North African countries, branch and leaf decoctions are prepared to cure intermittent fevers, arthritis and rheumatisms and used as a depurative, diuretic, hypoglycemiant and laxative [23,25]. The decoction of the aerial parts is administered against constipation, fever, and mycosis [28]. A leaf infusion is used as a hypoglycemic, digestive, and laxative as well as for bilious stimulation [24]
*Haplophyllum tuberculatum *(Forssk.) A. Juss. (PORUN - TTD59; TTF2064) RUTACEAE Additional file 11	*sézeret er rîh *(arab)	Aerial part	Laxative	In Egypt and Saharan territories, flowering and fruiting branches are used to cure gastro-intestinal affections, intermittent fevers, and rheumatisms. The plant is also an aphrodisiac and administered against eye and ear affections [23]
*Hedypnois cretica *(L.) Dum.Cours. (PORUN - TTF2312) ASTERACEAE	*sézeret er rîh *(arab)	Aerial part	Against meteorism and to cure haón (a kind of influence)	Medicinal uses not described for Maghreb and neighboring countries. Known as an edible plant in different Mediterranean countries [36,37]
*Hyoscyamus muticus *L. subsp. *falezlez *(Coss.) Maire (PORUN - TTF2315) SOLANACEAE	*gungot *(arab); *falezlez *(arab); *afahlehle *(temahac); *bathim*, *buthima*	Aerial part	The plant is severely poisonous. The extract of the aerial part mixed with butter is used externally against rheumatic affections	Known as medicinal plant in Marmarica [22]. In North Sahara, a potion is drunk as a tonic [29]. The oil macerate of leaves is topically used in Tassili N'ajjer against backache, muscular cramps, spasms, and palpitation anxiety as well as to treat eye inflammation and lice [28]
*Jateorhiza palmata *(Lam.) Miers (PORUN - TTD69; TTF2322) MENISPERMACEAE Additional file 12	*zarámba*	Roots	Infusion in orange water is given against cardiac affections	Its use is largely diffused in East and Central Africa as a bitter tonic, analgesic, and against diarrhea [38]
*Juniperus oxycedrus *L. s.l. (PORUN - TTF2327) CUPRESSACEAE	*taga *(Algeria)	Wood of the stem	Trunks are carbonized to give an oil (the cade oil) that is used against skin affections	In Tunisia and Morocco, the tar produced by the wood is antiparasitic and antiseptic for the skin [23]
*Launaea quercifolia *(Desf.) Pamp. (PORUN - TTF654) ASTERACEAE	*machinàn *(arab)	Aerial part	Eaten against rheumatic aches	Medicinal uses not described for Maghreb and neighboring countries. In Morocco, *L. arborescens *is used to cure diabetes and against nausea and skin troubles [21]
*Laurus nobilis *L. (PORUN - TTD47) LAURACEAE Additional file 13	*rénd, rhár *(arab)	Dried leaves	Medicinal plant (uses not described). Dried leaves are sold in the markets by herbalists	Leaf and fruit oil has a cosmetic application for face care. An infusion of leaves and fruits is administered for dental hygiene and to cure liver, pancreas and digestive diseases. The decoction of the same parts is given to treat rheumatic pains [24,25]
*Lawsonia inermis *L. (PORUN - TTF1798) LYTHRACEAE	*hénna *(arab); *alen *(berber); *anella *(temahac)	Aerial part	Astringent, vulnerary, used against dandruff and chilblains. Abortive	Leaves are antiseptic and astringent as well as used against eye affections and in the preparation of antirheumatic liniments. Flowers have insecticide properties [23]. Leaves powdered and mixed with water are applied as a hair tonic or mixed with lime juice as an emetic [24]. Also administered against gastric ulcer and kidney stones [25]
*Lepidium sativum *L. s.l. (PORUN - TTD34; TTF852) BRASSICACEAE Additional file 14	*habb' rsciad *(arab); *carabau*, *tsc'uit *(Algeria)	Seeds	Sold in the markets and used to cure cough and asthma	In North Africa, the seeds, crushed with honey, or seed flour are administered against cough and pulmonary affections, delivery difficulties, heart tonic, revulsive and also in the cure of skin troubles. The leaves are considered tonic and effective in the prevention of scurvy; frequently used as a condiment [23,25]. The milk infusion of seeds is used to cure migraines. Seeds boiled in oil are used to treat diarrhea. Powdered seeds are externally applied for skin ulcers and warts. Seeds are also part of a preparation used in the treatment of sexual impotence [24]
*Marrubium alysson *L. (PORUN - TTF1423) *Marrubium vulgare *L. (PORUN - TTF1429) LAMIACEAE	*rúbia*, *róbia *(arab)	Aerial part	The powdered plant, mixed with oil, is used for rheumatism	Flowers have insecticide properties [23]. A leaf and stem decoction is used to treat intestinal pains, cough, and colds. Crushed leaves are used against ear pains [26]. An infusion of the aerial parts is used as an antipyretic, expectorant, antidiarrheal, tonic, bilious stimulation and for bronchitis and menstrual pains. The aerial part is also boiled with wine to obtain a syrup used as a stomachic [24,25]
*Matricaria aurea *(Loefl.) Sch. Bip. (PORUN - TTD25; TTF666) ASTERACEAE Additional file 15	*fleia *(arab); *greisa *(Cirenaica)	Flowering branches	The decoction is used against gastro-intestinal affections	In the Middle East, the plant is used as a substitute for *M. chamomilla *[39]
*Myrtus communis *L. s.l. (PORUN - TTD31) MYRTACEAE Additional file 16	*rehân*, *ghemmâm*, *gidra *(arab)	Leaves and flowers	The plant has many medicinal uses	The plant is sold in the Algiers and Rabat markets for use against diarrhea, gastro-intestinal disorders, asthma and other respiratory ailments. It is also topically applied for painful organs. The plant is also used in perfumery and cosmetics [23,25]. Leaves and buds are used against hemorrhoids and skin affections. Fruits are administered for the cure of ocular disorders. An infusion of the leaves is used as a mouthwash against gingivitis and in association with walnut and mulberry in the treatment of diabetes [21]. A leaf decoction is used against cardiac and intestinal affections [26]. A leaf infusion is used against pneumonia, diarrhea and to promote wound healing. For hair care, a mixture with *Lawsonia inermis *is locally applied [24]
*Nitraria retusa *(Forssk.) Asch. (PORUN - TTF2317) ZYGOPHYLLACEAE	*gárdegh *(arab); *atazzim *(temahac)	Leaves	Crushed leaves are put in hot water and then used as a poultice against swellings	The leaves are used in Tunisia to reduce swellings, and the ashes are used to cure infected wounds [21]
*Olea europaea *L. s.l. (PORUN - TTF1811) OLEACEAE	*zêt, zêt ez zitûna*	Oil	Excipient of many plant drugs	A leaf decoction is used against nervous troubles and as an anthelmintic. Powdered leaves are used in the treatment of diabetes; burnt leaves are used to cure eye affections. Olive oil is administered to cure dry cough and grippe [26]. In Morocco, it is used for mouth hygiene, stomach pains, intestinal diseases, and diabetes [24,25]
*Opuntia ficus-indica *(L.) Mill. (PORUN - TTF2329) CACTACEAE	*híndi *(arab)	Flowers	Medicinal properties (not described)	The powdered flower is used against stomach disorders [26]. An infusion of flowers is administered to stop diarrhea and as a diuretic. Flowers are also part of a preparation used as a calefacient [24]. In Morocco, it is also administered to cure bladder, kidney and uterus infections [25]
*Orchis mascula *(L.) L. s.l. (PORUN - TTF2326) ORCHIDACEAE	*sahalep*	Whole plant	Used in the preparation of a medicinal powder	No medicinal use reported for Maghreb or Sahara
*Origanum majorana *L. (PORUN - TTD41) LAMIACEAE Additional file 17	*mardgúscia *(arab)	Aerial part	Against dysentery	In the Algiers market, the plant is sold to cure eye affections [23] In Morocco, the infusion of branches is used to treat chills, fever, cough, and flatulence [25,26]
*Origanum vulgare *L. s.l. (PORUN - TTF2303) LAMIACEAE		Flowers	Flowers have medicinal properties	In Tunisia and Algeria, the leaves and flowering branches are used as a stimulant [23]
*Paliurus spina-christi *Mill. (PORUN - TTF2321) RHAMNACEAE	*sédr*, *zegregh*, *ennab*, *corna *(arab); *abaga*, *labacat *(temahac); *magaria*, *cussulu *(Algeria)	Young shoots Leaves	Anthelmintic	Medicinal uses not reported in Maghreb and neighboring regions. Used in the East Mediterranean region to treat respiratory, circulatory and gastro-intestinal disorders [40]
*Paronychia argentea *Lam. (PORUN - TTF2301) PARONYCHIACEAE	*theia el arab, theia el gebel *(Algeria)	Aerial part	Infusion used against dysentery	Used as an antidiabetic in the East Mediterranean [41]
*Peganum harmala *L. (PORUN - TTD35; TTF2067) ZYGOPHYLLACEAE Additional file 18	*hármal *(arab); *bender tifli*, *bender tifîn *(tuaregh)	Seeds	Oil is used against headaches; burnt against mental diseases	The plant is sold in North African markets and used for a large number of affections. In Sudan, it is used against inflammations of the head, face and mouth as well as against headache and sinusitis [31]. The leaf decoction is used against high blood pressure and hemorrhoids [21]. In Tassili N'ajjer (Algeria), a decoction of seeds is taken to treat a large number of affections, ranging from skin diseases to nervous disorders, including anxiety. Also used to treat diabetes, helminthiasis and jaundice. The seed decoction is externally applied against tumors, eczema and lice [28]. Similar uses are also reported for Morocco and the Northern Sahara [25,29]. Powdered seeds of barley and *P. harmala *are topically applied against rheumatism [21]
*Periploca angustifolia *Labill. (PORUN - TTF2306) APOCYNACEAE	*sinuâc, teborac, arac *(arab); *têhac *(temahac); *chigu *(near Tchad)	Dried leaves Fruits	A decoction of dried leaves is used against syphilis mixed with a substance called ras el hânut A violet, pungent beverage is a slight laxative	The seed decoction is used as a local analgesic and antirheumatic [23]. In Tassili N'ajjer (Algeria), the decoction of seeds or the aerial part of the plant can be drunk as an abortive and to cure diabetes. Externally, used to treat rheumatism and various pains [28]. Fodder appreciated in arid areas, eaten by camels, sheep and goats
*Phoenix dactylifera *L. (PORUN - TTF2323) ARECACEAE	*na'hla *(arab); *tezdit *(berber); *tazzeit*, *tazeit *(temahac); *zui *(Augila)	Fruits	The fruits are used to make an alcoholic beverage to which is attributed medicinal properties.	In North African countries, the wood of the palm is used as toothbrush. Dates are used as against ulcers of the genital organs and as a diuretic, laxative, and tonic [23]
*Piper retrofractum *Vahl (PORUN - TTD19) PIPERACEAE Additional file 19	*dahr el filfil*	Flowers	Imported in Libya from Sudan. When mixed with honey is used as an aphrodisiac	The plant is imported from Asian countries. In Morocco, the plant is known for its aphrodisiac, calefacient and magic properties [25]
*Pistacia atlantica *Desf. (PORUN - TTD3; TTF16) ANACARDIACEAE Additional file 20	*batúm *(arab); *tizert *(berber)	Fruits	Sold in the markets. It is chewed against respiratory affections. The oil has similar properties	Leaves are used against skin affections. Fruits are used to season dates. Used for tanning [23]. In Marmarica, it is used as fuel, grazing and medicinal plants [22]. Burnt leaves are used in a poultice against eye affections [26]. In Morocco, fruits are administered against stomach-ache, whereas the gall is used for cosmetic applications, against fever and stomach diseases [25]
*Plantago afra *L. s.l. (PORUN - TTF2324) PLANTAGINACEAE	*anàm, nenàm *(arab)	Whole plant	Vulnerary. Powdered and dried is administered in topical applications	In Marmarica, used as medicinal plant [22]. In North African countries, used against metabolic disorders, gastro-intestinal affections, hemorrhoids, skin diseases, urinary tract disorders, and venereal diseases. Fresh leaves are applied topically for poison ivy, insect bites and stings [21]
*Punica granatum *L. (PORUN - TTD37) PUNICACEAE Additional file 21	*rummân, rummâna *(arab); *taarmunt, armun *(fruit) (berber); *tarrumant *(temahac)	Flowers	Flowers are medicinal. Seeds are tonic and aphrodisiac	In Egypt, fruit peels are sold as an astringent; dried powdered peel is sold in Morocco as an antidiabetic, antidiarrheal, antiseptic, antiulcerous, astringent, and hemostatic as well as against gastro-intestinal and gynecological disorders and for cleansing the teeth [23-26]
*Ricinus communis *L. s.l. (PORUN - TTD14) RUTACEAE Additional file 22	*chèrua *(arab)	Seeds	The water infusion is a laxative (also chewed seeds). Leaves are used in the preparation of poultices	Known as a medicinal plant in Marmarica [22]. In Central Sahara, the seed decoction is used against fevers and headaches or externally applied to cure trachoma, aphthae, and hair loss [28]. A decoction is administered to treat cow jaundice [24]. In Sudan, the fresh leaves are rubbed on the head to relieve headache or on the legs against swellings [30]
*Rosa damascena *Mill. (PORUN - TTF2325) ROSACEAE	*uárd *(arab)	Rose buds	The herbalists in Tripoli sell dried rose buds for medicinal purposes (sciús el uárd)	Dried flower buds are used against headache, stomach pains, toothaches, and as laxative and hair tonic are sold in North African markets [23,25]. Used also in the cure of numerous affection of the eyes and ears [23] The flower infusion is used as a laxative. A mixture with other plants is applied externally [24]
*Rosmarinus officinalis *L. (PORUN - TTD16; TTF1441) LAMIACEAE Additional file 23	*clíl *(arab); *uzbir*, *uzuer *(berber)	Leaves	The infusion is used against cough; ground dried leaves in oil are vulnerary	The leaf decoction is administered against intestinal parasites and rheumatism in Central Sahara [26] as well as an emmenagogue, spasmolytic against gastro-intestinal and liver disorders, diuretic, carminative and sedative in Morocco [25]. A leaf infusion is also prepared against tachycardia and as a cholagogue and vasopressor. Leaves are also externally applied against wrinkles, muscular pains and rheumatism and as a vulnerary [24]. In Tunisia, rosemary leaves are used as an antispasmodic for the digestive tracts and as a vermifuge. Dried leaves, ground and mixed with olive oil, are put on recent circumcision wounds [21]
*Ruta chalepensis *L. (PORUN - TTF2069) *Ruta *sp. (PORUN - TTD24) Additional file 24 *Ruta graveolens *L. (PORUN - TTF2309) RUTACEAE	*fgél*, *figél *(arab); *issîn *(temahac) *fgél, fesál *(arab)	Aerial part Aerial part	Sold in Tripoli market, and used against rheumatic affections and ecchymosis. The smell of the plant keeps the scorpions away from houses In Cyrenaica, a potion made with this plant is administered to newborns as a tonic	In Rabat Market, the plant is sold for use against nervous affections, and in Algiers markets, for use against vomiting and fevers of children and babies [23]. Washing with crushed leaves is used against ear pains, and leaves are smoked to keep away bad spirits [26]. In Morocco, the aerial parts are used as an abortive, for intestinal and hepatic diseases, male sterility, and vitiligo [25]. Also known as a vulnerary, emmenagogue, and spasmodic; the fresh plant is used as a scorpion and insect repellent. Leaves and seeds, boiled in olive oil, are rubbed on the skin to treat rheumatism and swellings [21]
*Salvia aegyptiaca *L. (PORUN - TTF1370) LAMIACEAE	*ra'al, sezeret el rházel *(arab, ex Muschler); *safsaf *(tuaregh)	1. aerial part; 2. leaves	The infusion is digestive. The leaves are kept in the nose to give a fresh sensation	In Marmarica, it is used as grazing plant [22]. In Tassili N'ajjer (Algeria), seeds are topically applied as an eye antiseptic. The infusion of the aerial part is a febrifuge, painkiller, and antispasmoic. Also used to treat digestive troubles and infected wounds [28]
*Schoenocaulon officinale *(Schltdl. et Cham.) A. Gray ex Benth. (PORUN - TTF2311) MELANTHIACEAE	*duá ghémel*	Seeds	Against lice	Used as an insecticide in North Africa and on other continents [42]
*Smyrnium olusatrum *L. (PORUN - TTF2193) APIACEAE	*calch *(arab)	Aerial part	Abortive	The genus *Smyrnium *is included among the medicinal plants of Morocco and is mainly used as a calefacient [25,43]; the fruits are ground to powder and put in water for a cold in the chest. The decoction is recommended for headaches [44]
*Tanacetum parthenium *(L.) Sch. Bip. (PORUN - TTF2332; TTD63) ASTERACEAE Additional file 25	*usciach*	Gum resin Roots	Imported from Marseille and sold in the markets. It is dissolved in vinegar against contusions or to cure furuncles Medicinal properties (not described)	Used against fever, rheumatoid arthritis and migraines in the popular medicine of Africa, Europe and America [45]
*Teucrium polium *L. s.l. (PORUN - TTD65; TTF1389) LAMIACEAE Additional file 26	*giaád*, *zaád *(arab); *techmezzutin *(tuaregh)	Aerial part	Gastro-intestinal affections	Used in North Africa against dysmenorrhea [21]. In Morocco, the aerial parts are used against chill, edema, live pain and for blood cleansing [25]
*Thapsia garganica *L. s.l. (PORUN - TTF2196) BRASSICACEAE	*drias *(arab)	Inflorescence	Vulnerary	The infusion of the aerial parts is used against cough and rheumatic pains. The root infusion is used to treat liver and bladder diseases, and an oil is externally applied for swelling and wrinkles [24]
*Thymus capitatus *(L.) Hoffmanns. et Link (PORUN - TTD70; TTF1401) Additional file 27 *Thymus mastigophorus *Lacaita (PORUN - TTF2328) LAMIACEAE	*zaátar *(arab); *sótar *(berber) *zaátar *(arab); *arrar*? (berber)	Leaves and floral shoots	A cold infusion against cough	Eaten as vegetable in Marmarica and also known as medicinal plant [22]. In Libya, the plant is used for coughs, as a tonic, and against skin affections [23]
*Trigonella foenum-graecum *L. (PORUN - TTF1596) FABACEAE	*hélba *(arab)	Seeds	Against cough, febrifuge	Cultivated and naturalized in North Africa [23]. The seed decoction is used for uterine pains. Boiled seeds are also ingested as a hypoglycemic, while powdered seed mixed with water are externally applied as a hair tonic [24]. In Morocco, seeds are considered to be blood cleansing and an aortic-palpitation reconstituant [25]. In Sudan, it is administered against rheumatism and dysentery as well as for cleaning the blood. It is also reputed as a lactagogue [31]. In Tasili N'ajjar (Algeria), the decoction of the aerial parts or seeds are used to cure diabetes, clean the blood, and as a tonic and an analeptic [28]. Seed maceration is effective to treat diabetes, scurvy, and digestive troubles [26]
*Zingiber officinale *Roscoe (PORUN - TTF2330) ZINGIBERACEAE	*zéngibil, schéngibil*	Roots	Aphrodisiac	Used in North Africa for a wide range of affections [23]. In Sudan, used for colds and rheumatism as well as to treat pneumonia [31]
*Evernia furfuracea *(L.) Mann (PORUN - TTD56) PARMELIACEAE Additional file 28	*scíba*	Fragments of thallus	When mixed with other species of lichens (see below), it is used to prepare medicinal decoctions	Medicinal uses not reported for Maghreb and neighboring regions
*Ramalina calicaris *(L.) Fr. (PORUN - TTD53) RAMALINACEAE Additional file 29	*scíba*	Fragments of thallus	See *Evernia furfuracea*	Medicinal uses not reported for Maghreb and neighboring regions
*Ramalina farinacea *(L.) Ach. (PORUN - TTD54) RAMALINACEAE Additional file 29	*scíba*	Fragments of thallus	See *Evernia furfuracea*	Medicinal uses not reported for Maghreb and neighboring regions
*Usnea plicata *(L.) Fries (PORUN - TTD55) USNEACEAE Additional file 30		Fragments of thallus	See *Evernia furfuracea*	Medicinal uses not reported for Maghreb and neighboring regions

The occurrence of? after a local name of a plant is a Trotter indication.

Trotter collected his ethnobotanical data mainly during the first expedition (1912). The provenance of the information is not always indicated, but we are far from the present standards of ethnobotanical investigations. Frequently, a single or very few informants are the source of the data, and, in some cases, Trotter reported only that the plant was used as a medicine, without describing the therapeutic applications. Notwithstanding these limitations, a not-irrelevant body of knowledge was assembled.

Data are related to about 70 species of flowering plants and to 4 lichens. The plants are mainly from Mediterranean or Sub-Saharan habitats, with a slight prevalence for the former. The plants belong to 37 different families; Lamiaceae was the most cited family with 10 accessions, followed by Asteraceae with 9. The prevalence of these families has been reported in the conclusive report of the Rubia project on medicinal plants in Mediterranean area [46]. The authors suggest that this could be because the plants belonging to these families are easily recognizable by the morphology of the flowers and due to their aromas and flavors.

Generally, the aerial parts of the plants are the most frequently used (28 species), followed by leaves (15 species), flowers and seeds (9 species), fruits (7 species) and hypogean organs (roots, rhizomes, tubers: 5 species).

Plants were generally processed in very simple ways: an infusion or decoction of the plants were prepared and orally administered or used for topical applications. A wide range of affections was treated, ranging from mental disorders to skin affections. All the organs of human body were considered, but the pathologies of the gastro-intestinal tract, respiratory system and those related to traumatic accidents (contusions, swellings, burnings, wounds) were the most frequently mentioned.

Trotter described a decoction, named *sciba*, made with three different lichens belonging to the genera *Evernia *and *Ramalina*. The species mentioned are found worldwide and largely used for dyeing [47], but the species are also used in perfumery [48] and in traditional medicine of numerous countries, due to the presence of active compounds, as usnic acid and atranorin [49]. Trotter did not indicate the therapeutic applications for this decoction. With a similar name, al-sheba, the lichen *Parmotrema tinctorium *is sold as food spice in India [50]. As far we know, there are no other reports dealing with this preparation in the Maghreb countries.

**Figure 7:**
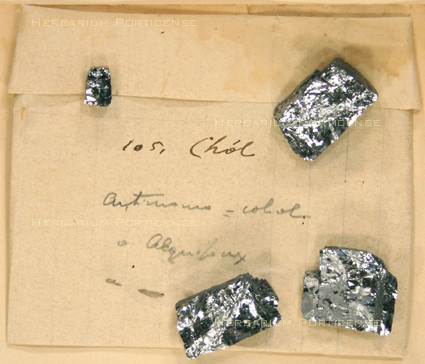
**Antimonium (PORUN - TTD76), Tripolitania Trotter collection Drug section**.

**Figure 8:**
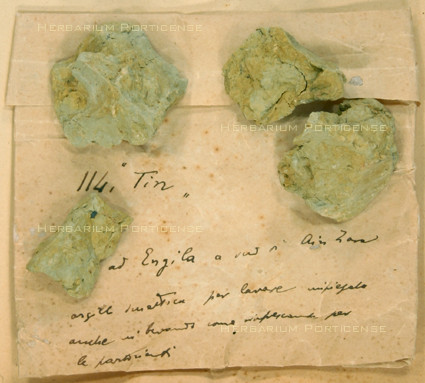
**Clay (PORUN - TTD77), Tripolitania Trotter collection Drug section**.

Overall, 75 plants (including the four lichens previously mentioned) were also collected as drugs by Trotter; 30 of these had medicinal properties. Two mineral samples (antimony and clay) were also held in the drug collection (Figure 7 and 8); they were used in topical wound treatment by numerous ancient and primitive societies [51]. The drugs were mainly sold in the Tripoli market, but also in local markets spread along Tripolitania. Libya is located in the middle of Mediterranean and was an important crossroad for trade in ancient times. The Libyan towns established commercial relationships with countries of all three continents, Africa, Asia and Europe. The town of Cyrene was a prime center for the export of the medicinal herb called silphium, one of the essential commodities of the Mediterranean region in classical era [52]. A late echo of the ancient flourishing trades was still present in the drugs found by Trotter at the beginning of the 20th century. He collected drugs that are, to a large extent, of Mediterranean origin, and are currently traded in Mediterranean region. Drugs from herbs such as *Ajuga iva *or *Artemisia arborescens *or those belonging to the genera of Lamiaceae, listed in Table 1, are commonly found in the markets along the Mediterranean, from Moroccan bazaars [44] to the herbal shops of Turkey [40] and Greece [53]. In contrast, some drugs were of Asiatic provenance, such as *Alpinia officinarum *[54] and *Piper retrofractum *[55], whereas others, such as *Tanacethum parthenium*, came mainly from Europe [56], suggesting that the ancient trade routes from Asia and Europe to North Africa were still being used a century ago. Few drugs were produced from indigenous plants; perhaps the most interesting case is that of *Ferula marmarica*, a plant native to some Libyan regions [34], which was used in classical times to produce the ammoniac gum [35], to which Dioscorides attributed relevant therapeutic roles ranging from anti-inflammatory to digestive and painkiller [57].

## Conclusion

The Trotter Collection can represent a useful tool for current ethnopharmacological research in Libya and neighboring countries. It is known that about 80% of the African population presently rely on traditional forms of health care, but it is not easy to document continuity and changes in therapeutic approaches. The information collected by Trotter contributes to filling this gap, enabling us to trace the use of plant utilization in Libyan folk medicine over the last century. A comparison with the recent ethnopharmacological research in Maghreb reveals a high correspondence; almost all of the plants cited by Trotter are still used in the folk medicine of at least one of the North African countries, and therapeutic uses of each plant appear consistent over that time.

The results of this study, although based on information that needs to be confirmed by current methodologies, seem to suggest that it is possible to find a core of a shared popular medicine along the African Coast of the Mediterranean Sea, probably due to climatic, cultural and linguistic continuity.

## Competing interests

The authors declare that they have no competing interests.

## Authors' contributions

The authors contributed equally to this paper. All authors read and approved the final manuscript.

## Supplementary Material

Additional file 1 ***Ajuga iva *(L.) Schreb**. **(PORUN - TTD52), aerial part.** Click here for file

Additional file 2 ***Alpinia officinarum *Hance (PORUN - TTD17), rhizome**. Click here for file

Additional file 3 ***Artemisia arborescens *L. (PORUN - TTD51), young shoots, flowers and leaves**. Click here for file

Additional file 4 ***Boswellia sacra *Flüeckiger (PORUN - TTD39), resin of stem**. Click here for file

Additional file 5 ***Capparis orientalis *Veillard in Duh**.** (PORUN - TTD7), leaves.** Click here for file

Additional file 6 ***Cuminum cyminum *L. (PORUN - TTD9), fruits**. Click here for file

Additional file 7 ***Cupressus sempervirens *L. (PORUN - TTD60), cones and seeds**. Click here for file

Additional file 8 ***Cymbopogon schoenanthus *(L.) Spreng. s.l**.** (PORUN - TTD79), basal part of the plant.** Click here for file

Additional file 9 ***Elettaria cardamomum *(L.) Maton (PORUN - TTD8), fruits**. Click here for file

Additional file 10 ***Ferula marmarica *Asch. et Schweinf**. **(PORUN - TTD22), gum resin.** Click here for file

Additional file 11 ***Haplophyllum tuberculatum *(Forssk.) A. Juss**. **(PORUN - TTD59), aerial part.** Click here for file

Additional file 12 ***Jateorhiza palmata *(Lam.) Miers (PORUN - TTD69), roots**. Click here for file

Additional file 13 ***Laurus nobilis *L. (PORUN - TTD47), leaves**. Click here for file

Additional file 14 ***Lepidium sativum *L. s.l. (PORUN - TTD34), seeds**. Click here for file

Additional file 15 ***Matricaria aurea *(Loefl.) Sch. Bip. (PORUN - TTD25), flowering branches and leaves**. Click here for file

Additional file 16 ***Myrtus communis *L. s.l. (PORUN - TTD31), leaves and flowers**. Click here for file

Additional file 17 ***Origanum majorana *L. (PORUN - TTD41), aerial part**. Click here for file

Additional file 18 ***Peganum harmala *L. (PORUN - TTD35), seeds**. Click here for file

Additional file 19 ***Piper retrofractum *Vahl (PORUN - TTD19), flowers**. Click here for file

Additional file 20 ***Pistacia atlantica *Desf**.** (PORUN - TTD3), fruits and gall-nut.** Click here for file

Additional file 21 ***Punica granatum *L. (PORUN - TTD37), flowers**. Click here for file

Additional file 22 ***Ricinus communis *L. s.l. (PORUN - TTD14), seeds**. Click here for file

Additional file 23 ***Rosmarinus officinalis *L. (PORUN - TTD16), leaves**. Click here for file

Additional file 24 ***Ruta *sp. (PORUN - TTD24), aerial part**. Click here for file

Additional file 25 ***Tanacetum parthenium *(L.) Sch. Bip. (PORUN - TTD63), roots**. Click here for file

Additional file 26 ***Teucrium polium *L. s.l. (PORUN - TTD65), aerial part**. Click here for file

Additional file 27 ***Thymus capitatus *(L.) Hoffmanns**. et Link **(PORUN - TTD70), leaves and floral shoots.** Click here for file

Additional file 28 ***Evernia furfuracea *(L.) Mann (PORUN - TTD56), thallus**. Click here for file

Additional file 29 ***Ramalina calicaris *(L.) Fr. (PORUN - TTD53) and *Ramalina farinacea *(L.) Ach. (PORUN - TTD54), thallus**. Click here for file

Additional file 30 ***Usnea plicata *(L.) Fries (PORUN - TTD55), thallus**. Click here for file
