# Supplementary figures and images for: A forgotten collection: the Libyan ethnobotanical exhibits (1912-14) by A. Trotter at the Museum O. Comes at the University Federico II in Naples, Italy
Source: J Ethnobiol Ethnomed. 2012 Jan 21;8:4. doi: 10.1186/1746-4269-8-4 (PMC3295647; doi:10.1186/1746-4269-8-4)

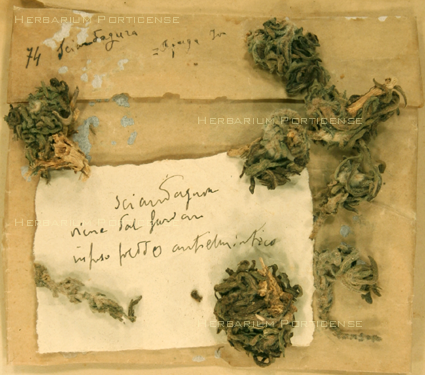

Supplement: Additional file 1 — Ajuga iva (L.) Schreb. (PORUN - TTD52), aerial part. [file 1746-4269-8-4-S1.TIFF]

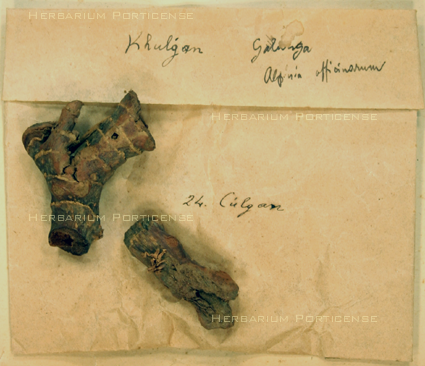

Supplement: Additional file 2 — Alpinia officinarum Hance (PORUN - TTD17), rhizome. [file 1746-4269-8-4-S2.TIFF]

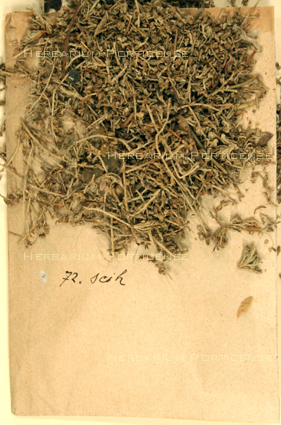

Supplement: Additional file 3 — Artemisia arborescens L. (PORUN - TTD51), young shoots, flowers and leaves. [file 1746-4269-8-4-S3.TIFF]

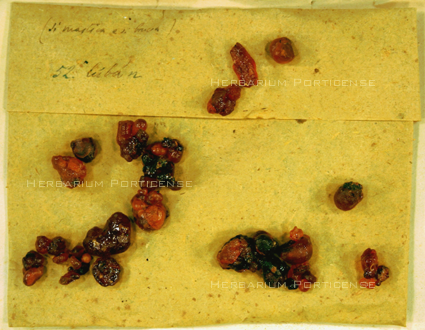

Supplement: Additional file 4 — Boswellia sacra Flüeckiger (PORUN - TTD39), resin of stem. [file 1746-4269-8-4-S4.TIFF]

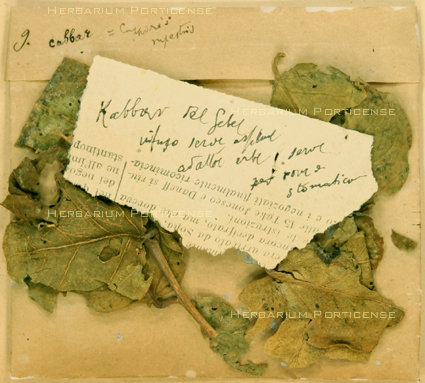

Supplement: Additional file 5 — Capparis orientalis Veillard in Duh. (PORUN - TTD7), leaves. [file 1746-4269-8-4-S5.TIFF]

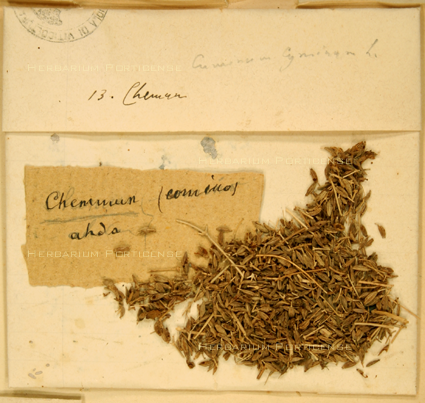

Supplement: Additional file 6 — Cuminum cyminum L. (PORUN - TTD9), fruits. [file 1746-4269-8-4-S6.TIFF]

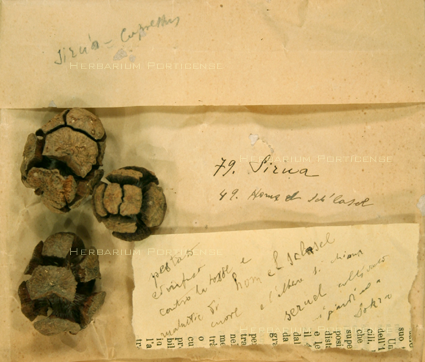

Supplement: Additional file 7 — Cupressus sempervirens L. (PORUN - TTD60), cones and seeds. [file 1746-4269-8-4-S7.TIFF]

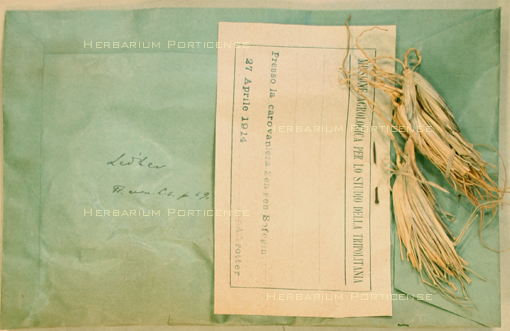

Supplement: Additional file 8 — Cymbopogon schoenanthus (L.) Spreng. s.l. (PORUN - TTD79), basal part of the plant. [file 1746-4269-8-4-S8.TIFF]

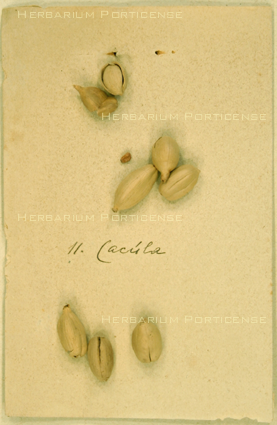

Supplement: Additional file 9 — Elettaria cardamomum (L.) Maton (PORUN - TTD8), fruits. [file 1746-4269-8-4-S9.TIFF]

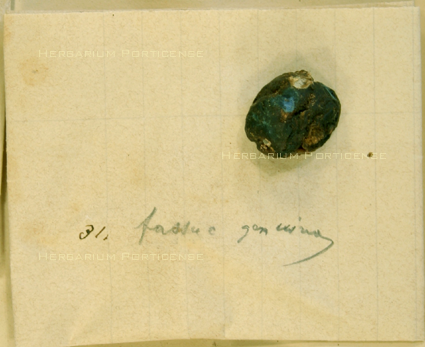

Supplement: Additional file 10 — Ferula marmarica Asch. et Schweinf. (PORUN - TTD22), gum resin. [file 1746-4269-8-4-S10.TIFF]

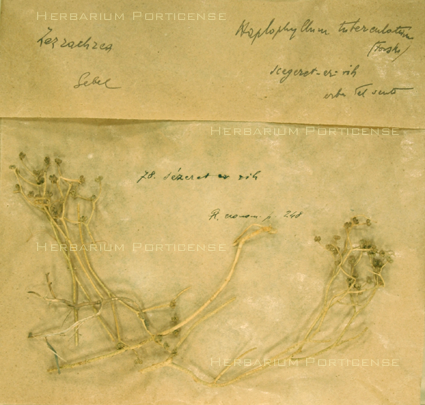

Supplement: Additional file 11 — Haplophyllum tuberculatum (Forssk.) A. Juss. (PORUN - TTD59), aerial part. [file 1746-4269-8-4-S11.TIFF]

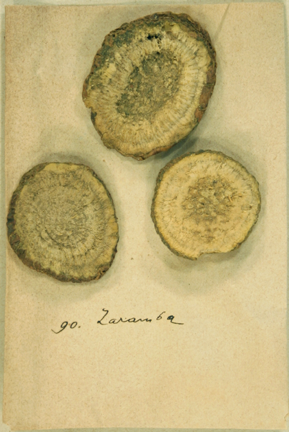

Supplement: Additional file 12 — Jateorhiza palmata (Lam.) Miers (PORUN - TTD69), roots. [file 1746-4269-8-4-S12.TIFF]

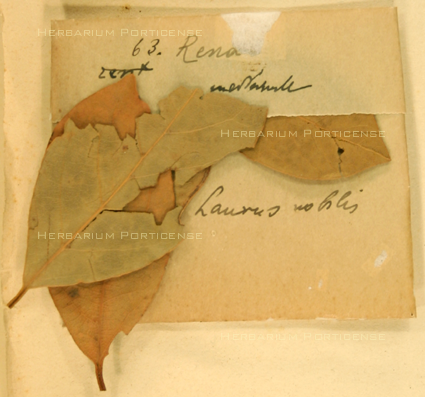

Supplement: Additional file 13 — Laurus nobilis L. (PORUN - TTD47), leaves. [file 1746-4269-8-4-S13.TIFF]

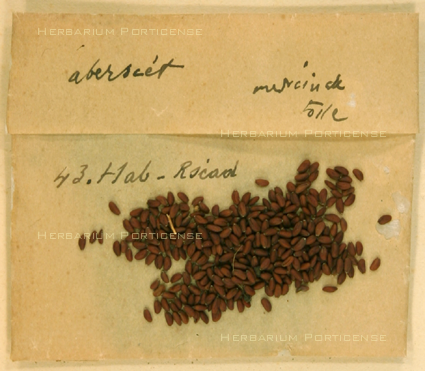

Supplement: Additional file 14 — Lepidium sativum L. s.l. (PORUN - TTD34), seeds. [file 1746-4269-8-4-S14.TIFF]

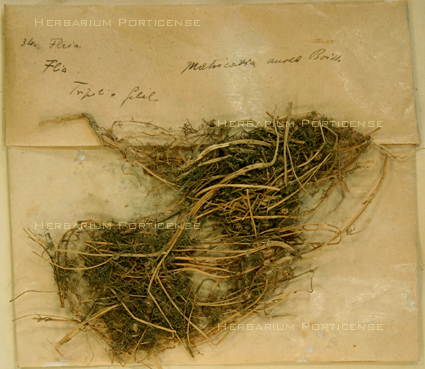

Supplement: Additional file 15 — Matricaria aurea (Loefl.) Sch. Bip. (PORUN - TTD25), flowering branches and leaves. [file 1746-4269-8-4-S15.TIFF]

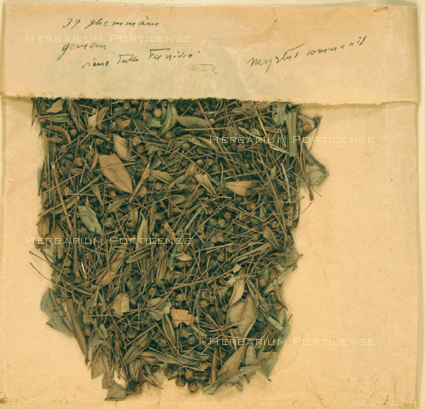

Supplement: Additional file 16 — Myrtus communis L. s.l. (PORUN - TTD31), leaves and flowers. [file 1746-4269-8-4-S16.TIFF]

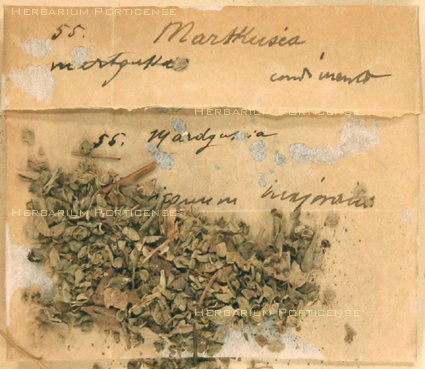

Supplement: Additional file 17 — Origanum majorana L. (PORUN - TTD41), aerial part. [file 1746-4269-8-4-S17.TIFF]

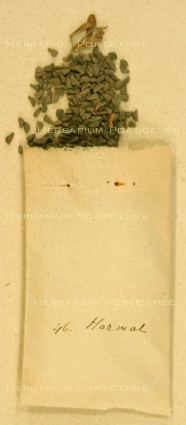

Supplement: Additional file 18 — Peganum harmala L. (PORUN - TTD35), seeds. [file 1746-4269-8-4-S18.TIFF]

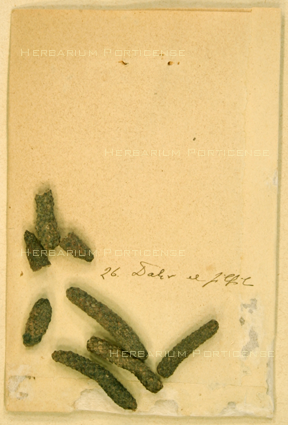

Supplement: Additional file 19 — Piper retrofractum Vahl (PORUN - TTD19), flowers. [file 1746-4269-8-4-S19.TIFF]

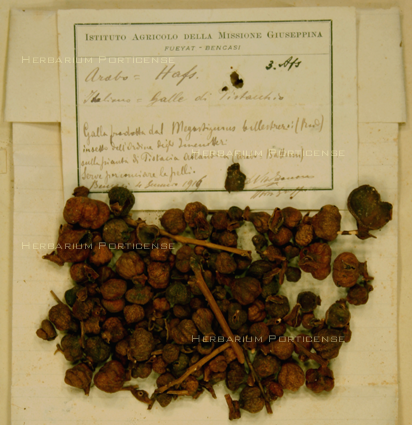

Supplement: Additional file 20 — Pistacia atlantica Desf. (PORUN - TTD3), fruits and gall-nut. [file 1746-4269-8-4-S20.TIFF]

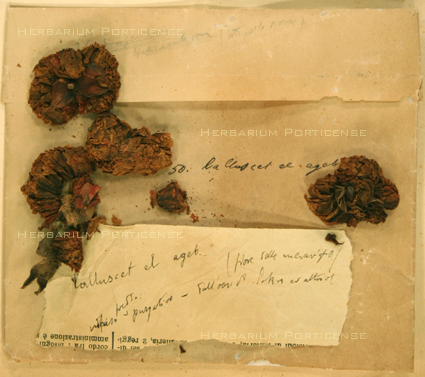

Supplement: Additional file 21 — Punica granatum L. (PORUN - TTD37), flowers. [file 1746-4269-8-4-S21.TIFF]

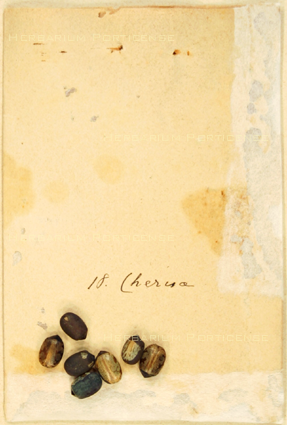

Supplement: Additional file 22 — Ricinus communis L. s.l. (PORUN - TTD14), seeds. [file 1746-4269-8-4-S22.TIFF]

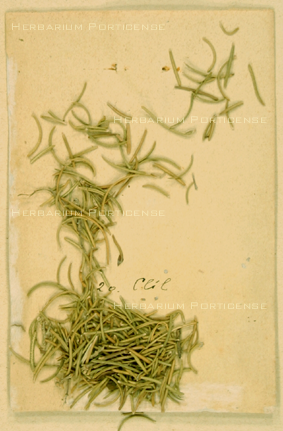

Supplement: Additional file 23 — Rosmarinus officinalis L. (PORUN - TTD16), leaves. [file 1746-4269-8-4-S23.TIFF]

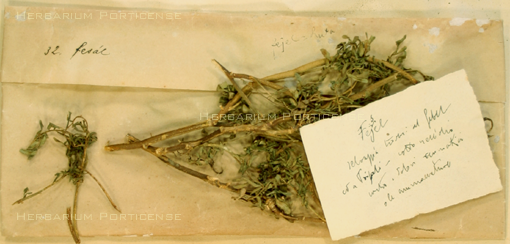

Supplement: Additional file 24 — Ruta sp. (PORUN - TTD24), aerial part. [file 1746-4269-8-4-S24.TIFF]

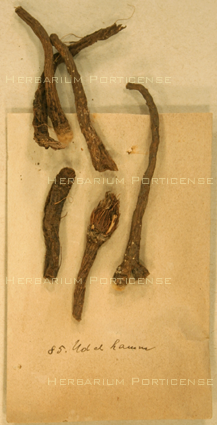

Supplement: Additional file 25 — Tanacetum parthenium (L.) Sch. Bip. (PORUN - TTD63), roots. [file 1746-4269-8-4-S25.TIFF]

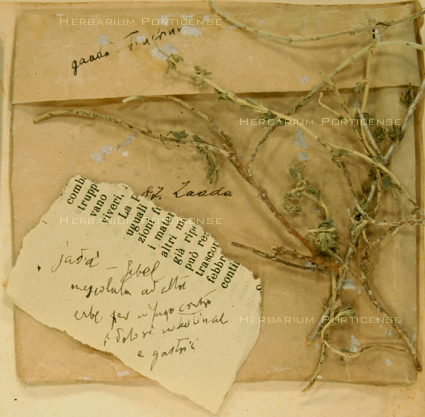

Supplement: Additional file 26 — Teucrium polium L. s.l. (PORUN - TTD65), aerial part. [file 1746-4269-8-4-S26.TIFF]

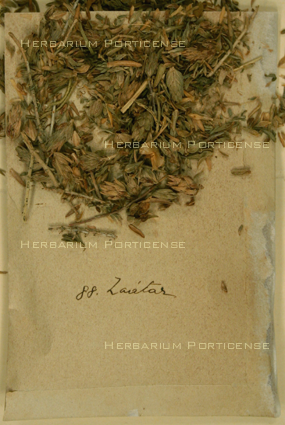

Supplement: Additional file 27 — Thymus capitatus (L.) Hoffmanns. et Link (PORUN - TTD70), leaves and floral shoots. [file 1746-4269-8-4-S27.TIFF]

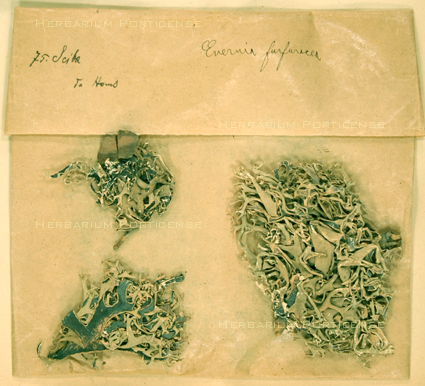

Supplement: Additional file 28 — Evernia furfuracea (L.) Mann (PORUN - TTD56), thallus. [file 1746-4269-8-4-S28.TIFF]

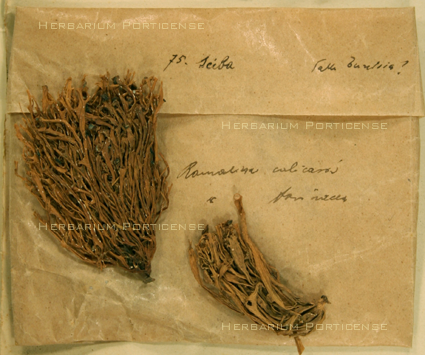

Supplement: Additional file 29 — Ramalina calicaris (L.) Fr. (PORUN - TTD53) and Ramalina farinacea (L.) Ach. (PORUN - TTD54), thallus. [file 1746-4269-8-4-S29.TIFF]

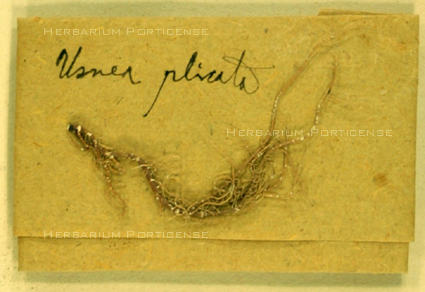

Supplement: Additional file 30 — Usnea plicata (L.) Fries (PORUN - TTD55), thallus. [file 1746-4269-8-4-S30.TIFF]
